# Combination of biomarker with clinical risk factors for prediction of severe acute kidney injury in critically ill patients

**DOI:** 10.1186/s12882-020-02202-z

**Published:** 2020-12-10

**Authors:** Lan Jia, Xiaohua Sheng, Anna Zamperetti, Yun Xie, Valentina Corradi, Shikha Chandel, Massimo De Cal, Diego Pomarè Montin, Carlotta Caprara, Claudio Ronco

**Affiliations:** 1 Department of Kidney Disease and Blood Purification, Institute of Urology & Key Laboratory of Tianjin, The Second Hospital of Tianjin Medical University, Tianjin, 300211 China; 2 International Renal Research Institute of Vicenza, San Bortolo Hospital, 36100 Vicenza, Italy; 3 Department of Nephrology, Shanghai Jiao Tong University Affiliated Sixth People’s Hospital, Shanghai, 200233 China; 4 Department of Nephrology, Xin Hua Hospital Affiliated to Shanghai Jiaotong University School of Medicine, Shanghai, 200092 China; 5 Center for Translational Research on Autoimmune and Allergic Diseases, University of Piemonte Orientale, 28100 Novara, Italy; 6 Department of Nephrology, Dialysis and Transplantation, San Bortolo Hospital, 36100 Vicenza, Italy

**Keywords:** Acute kidney injury, Clinical prediction, Intensive care, Risk factors, Insulin-like growth factor-binding protein 7, Tissue inhibitor of metalloproteinase-2

## Abstract

**Background:**

Acute kidney injury (AKI) occurs commonly in the intensive care unit (ICU). Insulin-like growth factor-binding protein 7 (IGFBP7) and tissue inhibitor of metalloproteinase-2 (TIMP-2), known as [TIMP-2] x [IGFBP7] (NephroCheck), have been identified as novel biomarkers for the prediction of AKI risk. However, the effective use of disease biomarkers is indispensable from an appropriate clinical context. We conducted a retrospective cohort study to find risk factors and assess the performance of the combination of NephroCheck with risk factors, so as to provide feasible information for AKI prediction.

**Methods:**

All patients who were admitted in the ICU (from June 2016 to July 2017) participated in the study. The primary outcome was the detection of severe AKI within the first 7 days after patients being admitted to the ICU. The predictors were separated into three categories: chronic risk factors, acute risk factors and biochemical indicators.

**Results:**

The study included 577 patients. 96 patients developed to severe AKI (16.6%) within 7 days. In addition to NephroCheck (+) (OR = 2.139, 95% CI (1.260–3.630), *P* = 0.005), age > 65 years (OR = 1.961, 95% CI (1.153–3.336), *P* = 0.013), CKD (OR = 2.573, 95% CI (1.319–5.018), *P* = 0.006) and PCT (+)(OR = 3.223, 95% CI (1.643–6.321), *P* = 0.001) were also the independent predictors of severe AKI within 7 days. Compared to NephroCheck (+) only (AUC = 0.66, 95% CI:0.60–0.72), the combination of NephroCheck (+) and risk factors (age > 65 years, CKD and PCT positive) (AUC = 0.75, 95% CI:0.70–0.81) led to a significant increase in the area under ROC curve for severe AKI prediction within 7 days.

**Conclusions:**

Although NephroCheck is an effective screening tool for recognizing high-risk patients, we found that combination with biomarker and risk factors (age > 65 years, CKD, procalcitonin positive) for risk assessment of AKI has the greatest significance to patients with uncertain disease trajectories.

## Background

Acute kidney injury (AKI) is a common clinical condition occurring in intensive care unit (ICU) patients and is confirmed as a strongly independent risk factor with high mortality. 50% of ICU patients will develop AKI and more than 20% of critically ill patients will develop to severe AKI in stage 2 and 3 (Kidney Disease: Improving Global Outcomes, KDIGO) [1]. However, AKI is usually unpredictable. For a large proportion of patients, the development of AKI has no obvious warnings or symptoms and remains clinically silent until a sudden drop of renal functions [2].

AKI could be identified by reduced urine output (urine volume < 0.5 mL/kg/h for 6 h) and increased serum creatinine (SCr) level (≥26.5 μmol/L within 48 h), however, it has been shown to be a lagging marker [3]. Owing to the limitations of SCr and urine output, many efforts have been made to find biomarkers that can be used as “kidney troponin”, which ideally predicts the severity and prognosis of AKI before markers of nephrological function change [4]. Therefore, many studies were conducted to discover and validate new AKI biomarkers. Until September 2014, following the publication of two multicenter ICU cohort studies, the combination of insulin-like growth factor-binding protein 7 (IGFBP7) and tissue inhibitor of metalloproteinase-2 (TIMP-2), known as [TIMP-2] x [IGFBP7] (NephroCheck), has been approved for marketing by the US Food and Drug Administration (FDA) [5–7]. This is the first biomarker used for AKI risk assessment which can help intensive care physicians and nephrologists make early predictions for AKI in intensive care settings, optimize the timing of resuscitation and promote supportive care for patients with AKI risk.

Recent research has focused on the use of biomarkers for AKI to recognize high-risk patients, however, most of these studies have not been integrated with clinical risk factors of AKI. The random and non-directional use of any biomarker will reduce its effectiveness [5, 6, 8, 9]. Basu et al. [10] have shown that combining clinical data with biomarkers can improve the accuracy of predicting severe AKI risk in pediatric ICU patients. The effective use of disease biomarkers is indispensable from an appropriate clinical context [11]. We hypothesized that the combination of the biomarker NephroCheck with risk factors would provide feasible information for the assessment of AKI and promote early intervention to improve clinical outcomes.

## Methods

### Study population

All patients (age ≥ 18 years old) who were admitted in the ICU of San Bortolo Hospital (Vicenza, Italy) from June 2016 to July 2017 participated in the study. End-stage renal disease (ESRD) patients, anuria patients and patients diagnosed with severe AKI (stage 2 and 3) were excluded. This study was approved by the Ethics Committee of San Bortolo Hospital, Vicenza, Italy (Comitato Etico provinciale aULSS 8 Vicenza) (Exp. number: 03/17). The clinical research was conducted according to the principles expressed in the *Declaration of Helsink*i.

### Study endpoint

The primary outcome was the detection of severe AKI (stage 2 and 3) within the first 7 days after patients being admitted to the ICU. Secondary outcomes included continuous renal replacement therapy (CRRT) initiation, ICU mortality, and length of stay (LOS). LOS means the duration of ICU stay.

### Data collection

Urine samples for measuring [TIMP-2] x [IGFBP7] concentrations were obtained and analyzed immediately following enrollment. The concentration of two proteins ([TIMP-2] and [IGFBP7]) was analyzed with the Vitros Platform (Ortho Clinical Diagnostics) using NephroCheck Kits (Astute Medical). Another point to be noted is that the test was acquired by the central laboratory at market price. In order to develop a new diagnostic tool, the hospital allocated a special budget. Other data were collected from hospital records, including demographics, anthropometry, admission diagnosis, comorbidities, simplified acute physiology score II (SAPS II) [12] on admission, mean arterial pressure (MAP) on admission, SCr levels on admission, lactate levels on admission and procalcitonin (PCT) levels on admission. A single SCr was recorded per day. In addition, data on CRRT, death and ICU discharge were recorded.

### Definitions

Severe AKI was defined as 2.0 or more multiplied by baseline SCr according to KDIGO consensus guidelines. NephroCheck > 0.3 (ng/ml)^2^/1000 was considered positive and a value of ≤0.3 (ng/ml)^2^/1000) was considered negative. PCT > 0.5 μg/l was considered positive and a value of ≤0.5 μg/l was considered negative.

### Risk factor profiling

By reviewing literature [13–17], the predictors we identified were separated into three categories: chronic risk factors, acute risk factors and biochemical indicators. Chronic risk factors included advanced age (age > 65 years), obesity (BMI > 30 kg/m^2^), hypertension, diabetes mellitus (DM), chronic kidney disease (CKD), lung disease and cardiovascular disease (CVD). Acute risk factors were sepsis, high-risk surgery, MAP< 70 mmHg, patients requiring vasopressors and mechanical ventilation. Biochemical indicators were SCr levels on admission, lactate levels on admission, Nephrocheck levels on admission and procalcitonin (PCT) levels on admission.

### Statistical analysis

The percentage was calculated for category variable. Continuous variables are described as medians (interquartile range). The categorical variables between the two groups were compared using Fisher’s exact test or chi-square test. The Mann-Whitney test was used in the comparison of two groups and the Kruskal-Wallis test was used in the comparison of three groups. In pretreatment step, variables were pre-screened using univariate logistic regression analysis. Once the univariate predictor of AKI is determined, then multivariate logistic regression is used to select variables, thus eliminating the collinearity and interaction of selected predictors. Comparison of the areas under the receiver operating characteristic (ROC) curve was made using the nonparametric method. *P* < 0.05 was considered statistically significant. Analysis was performed using SPSS Version 24.0 (IBM Corp, Somers, NY, USA).

## Results

### Study population

**Fig. 1:**
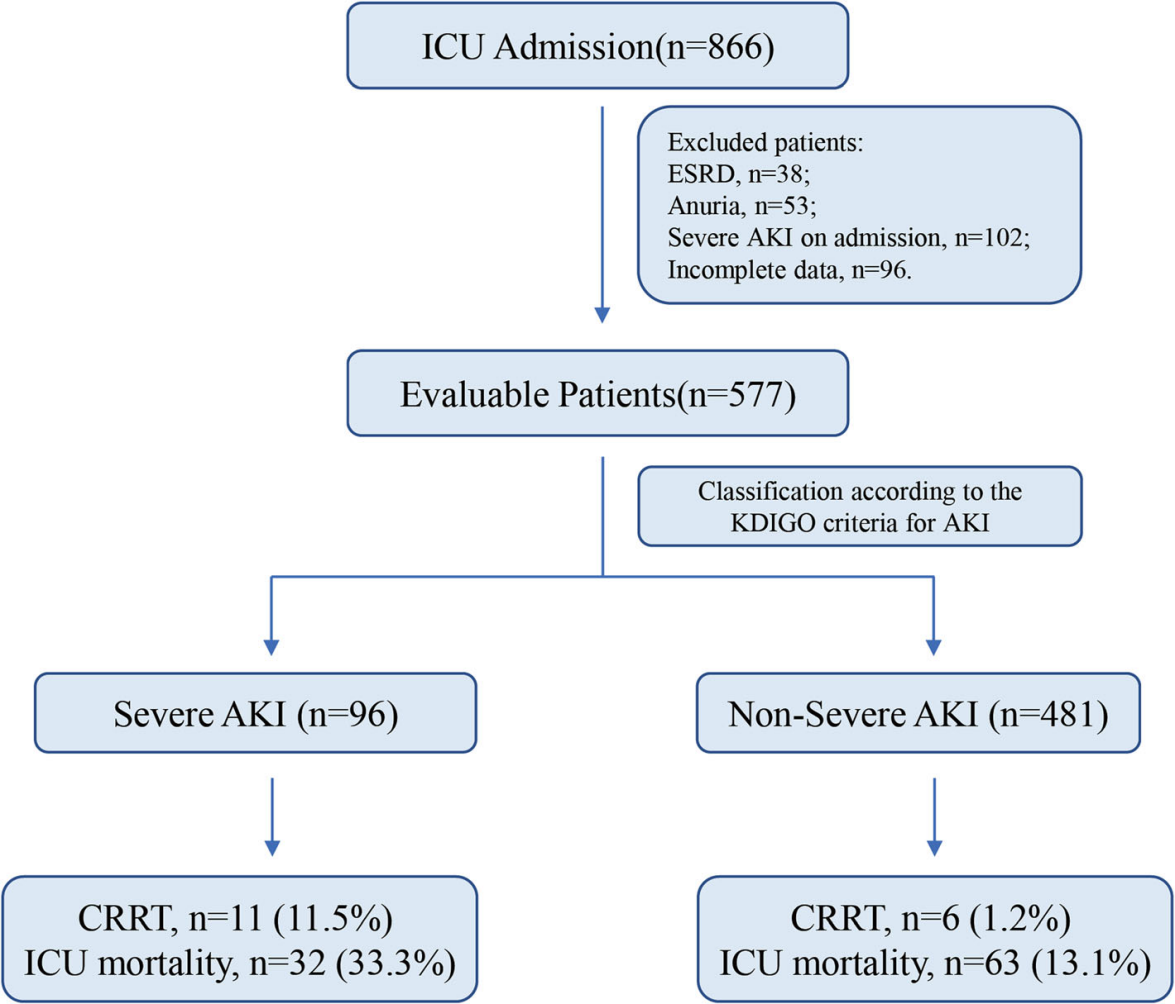
The flowchart of this study and the number of patients

In consecutive 866 adult patients (age ≥ 18 years) who underwent screening, 289 unsuitable patients were excluded. Therefore, the study included 577 patients and 96 of them developed to severe AKI (16.6%) within 7 days in the ICU. The flowchart of this study and the number of patients are as presented in Fig. 1. Admission diagnoses for these patients included surgery (14.7%), sepsis (10.6%), cardiovascular disease (11.8%), trauma (22.7%), respiratory diseases (8.8%), neurological diseases (21%) and other causes (10.4%).

### Patient characteristics

Patient characteristics are listed in Table 1. Severe AKI group enrolled 96 (16.6%) patients and non-severe AKI group enrolled 481 (83.4%) patients. Compared with patients from the non-severe AKI group, patients of severe AKI group were significantly older, had a higher body mass index and more CKD, sepsis and hypotension (MAP< 70 mmHg). ICU patients in severe AKI group also had a higher SCr level, lactate level, NephroCheck value and PCT level on admission.

**Table 1:** Baseline characteristics and outcomes of the study population by presence or absence of severe AKI within 7 days

Variable	Severe AKI	Non-Severe AKI	***P*** Value
N	96(16.6)	481(83.4)	
Age (years)	73(58–81)	67(51–77)	0.004
Male	64(66.7)	301(62.6)	0.448
BMI (kg/m^2^)	26.18(23.03–29.41)	24.8(22.86–27.68)	0.037
SAPSII	44(34–52)	38(27–50)	0.153
**Chronic risk factors**			
Age > 65 years	66(68.8)	256(53.2)	0.005
BMI > 30 kg/m^2^	21(21.9)	61(12.7)	0.039
Hypertension	48(50)	212(44.1)	0.49
DM	21(21.9)	66(13.7)	0.138
CKD	20(20.8)	39(8.1)	<0.001
Lung diseases	8(8.3)	43(8.9)	0.848
CVD	10(10.4)	58(12.1)	0.649
**Acute risk factors**			
Sepsis	20(20.8)	41(8.5)	<0.001
Surgery	18(18.8)	67(13.9)	0.224
Vasopressor	46(47.9)	179(37.2)	0.05
Mechanical ventilation	71(74)	329(68.4)	0.281
MAP< 70 mmHg	40(41.7)	109(22.7)	<0.001
**Biochemical indicators**			
Serum creatinine, admission (mg/dl)	1.08 (0.75–1.45)	0.83(0.65–1.08)	<0.001
Serum lactate, admission (mmol/l)	2.1(1.4–3.8)	1.6(1.2–2.6)	0.001
NephroCheck value, admission((ng/ml)^2^/1000)	0.66(0.23–2.49)	0.29(0.08–0.86)	<0.001
PCT, admission (ug/l)	1.19(0.28–6.81)	0.26(0.10–1.45)	<0.001
Nephrocheck (+)	69(71.9)	233(48.4)	<0.001
PCT (+)	54(56.3)	115(23.9)	<0.001
**Outcomes**			
CRRT	11(11.5)	6(1.2)	<0.001
Death	32(33.3)	63(13.1)	<0.001
LOS(d)	5(2–8)	3(2–7)	0.034

Data are expressed as n (%) or median (interquartile range)

*BMI* Body mass index, *SAPS II* Simplified acute physiology score II, *DM* Diabetes mellitus, *CKD* Chronic kidney disease, *CVD* Cardiovascular disease, *MAP* Mean arterial pressure, *PCT* Procalcitonin, *CRRT* Continuous renal replacement therapy, *LOS* Length of stay

### Severe AKI within seven days is associated with poor outcomes in ICU patients

Table 1 shows 11.5% of patients in severe AKI group and 1.2% of patients in non-severe AKI group required CRRT (*P* < 0.001). Severe AKI was also associated with ICU mortality. The mortality incidence was 33.3% in severe AKI group and 13.1% in non-severe AKI group respectively (*P* < 0.001). Severe AKI also increased LOS in ICU. LOS of severe AKI group and non-severe AKI group were 5 (2–8) and 3 (2–7), respectively (*P* = 0.034).

### Univariate variables associated with severe AKI within seven days

Table 2 provides a list of significant univariate variables associated with severe AKI within 7 days. The presence of hypertension, CVD, lung disease, high-risk surgery, mechanical ventilation and SAPSII cannot predict the development of AKI in our study. Among all chronic risk factors, age > 65 years, BMI > 30 kg/m^2^, DM and CKD could predict severe AKI, and the relative risk was 1.934 (95% CI(1.212–3.085,) *P* = 0.006), 1.887 (95% CI(1.085–3.282), *P* = 0.025), 1.748 (95% CI (1.009–3.027), *P* = 0.046), 2.982 (95% CI (1.651–5.388), *P* < 0.001), respectively. Among acute risk factors, sepsis and MAP< 70 mmHg could predict severe AKI, with a relative risk of 2.824 (95% CI (1.570–5.081), *P* = 0.001) and 2.431 (95% CI (1.537–3.845), *P* < 0.001). Among biochemical indicators, elevated SCr level was associated with a relative risk of 1.697 of developing severe AKI (95% CI (1.263–2.28), *P* < 0.001). With an increase of serum lactate concentration, the risk of developing severe AKI (OR = 1.115, 95% CI (1.036–1.199), *P* = 0.003) would be increased by 11.5%. In addition, NephroCheck (+) predicts the development of severe AKI with a relative risk of 2.72 (95% CI (1.684–4.394), *P* < 0.001). PCT (+) predicts the development of severe AKI with a relative risk of 4.883 (95% CI (2.625–9.084), *P* < 0.001).

**Table 2:** Logistic regression analysis for predictor of severe AKI within 7 days

Variable	Univariate	Multivariate
**Chronic risk factors**		
Age > 65 years	1.934 (1.212–3.085)	1.961(1.153–3.336)
BMI > 30 kg/m^2^	1.887 (1.085–3.282)	NS
DM	1.748 (1.009–3.027)	NS
CKD	2.982 (1.651–5.388)	2.573(1.319–5.018)
**Acute risk factors**		
Sepsis	2.824 (1.570–5.081)	NS
MAP< 70 mmHg	2.431 (1.537–3.845)	NS
**Biochemical indicators**		
Serum creatinine, admission (mg/dl)	1.697 (1.263–2.281)	NS
Serum lactate, admission (mmol/l)	1.115 (1.036–1.199)	NS
Nephrocheck (+)	2.720 (1.684–4.394)	2.139(1.260–3.630)
PCT (+)	4.883 (2.625–9.084)	3.223(1.643–6.321)

Data are expressed as odds ratio (95% CI). NS: Nonsignificant predictors

*BMI* Body mass index, *DM* Diabetes mellitus, *CKD* Chronic kidney disease, *MAP* Mean arterial pressure, *PCT* Procalcitonin

### Independent predictors of severe AKI within seven days

Multivariate logistic regression was performed with univariate variables related to severe AKI within 7 days. Following variable selection, four independent predictors, including age > 65 years (OR = 1.961, 95% CI (1.153–3.336), *P* = 0.013), CKD (OR = 2.573, 95% CI (1.319–5.018), *P* = 0.006), NephroCheck (+) on admission (OR = 2.139, 95% CI (1.260–3.630), *P* = 0.005) and PCT (+) on admission (OR = 3.223, 95% CI (1.643–6.321), *P* = 0.001) were used to predict the development of severe AKI (Table 2).

### NephroCheck level on admission was associated with incidence of severe AKI within seven days and its poor outcomes

**Fig. 2:**
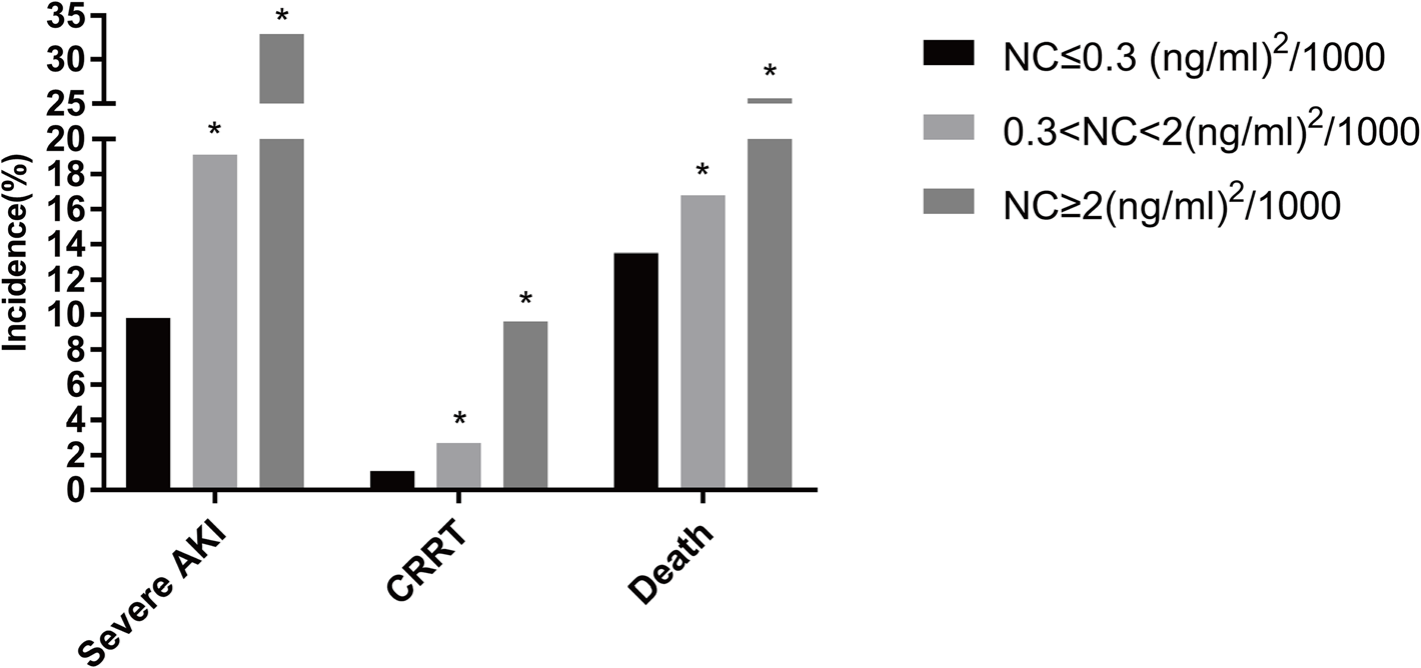
The graph shows that NephroCheck level on admission has a positive relationship with incidence of severe AKI within seven days and its poor outcomes. NC: NephroCheck. *P < 0.05

For 577 patients, 275 (47.7%) were NephroCheck≤0.3 (ng/ml)^2^/1000, 220 (38.1%) were NephroCheck (0.3–2) (ng/ml)^2^/1000 and 82 (14.2%) were NephroCheck≥2(ng/ml)^2^/1000. Severe AKI incidence within 7 days, CRRT initiation and ICU mortality were the highest in NephroCheck≥2(ng/ml)^2^/1000 group. The incidence of severe AKI within 7 days increased from 9.8% in NephroCheck ≤0.3 (ng/ml)^2^/1000 patients to 19.1% in NephroCheck (0.3–2) (ng/ml)^2^/1000 patients and 32.9% in NephroCheck≥2(ng/ml)^2^/1000 patients (compared with three groups, *P* < 0.001). The treatment of CRRT increased from 1.1% in NephroCheck ≤0.3 (ng/ml)^2^/1000 patients to 2.7% in NephroCheck (0.3–2) (ng/ml)^2^/1000 patients and 9.6% in NephroCheck≥2(ng/ml)^2^/1000 patients (compared with three groups, *P* < 0.001). ICU mortality increased from 13.5% in NC (−) patients to 16.8% in NephroCheck (0.3–2) (ng/ml)^2^/1000 patients and 25.6% in NephroCheck≥2(ng/ml)^2^/1000 patients (compared with three groups, *P* = 0.033) (Fig. 2).

### Incorporation of risk factors augments the predictive performance of the NephroCheck

**Fig. 3:**
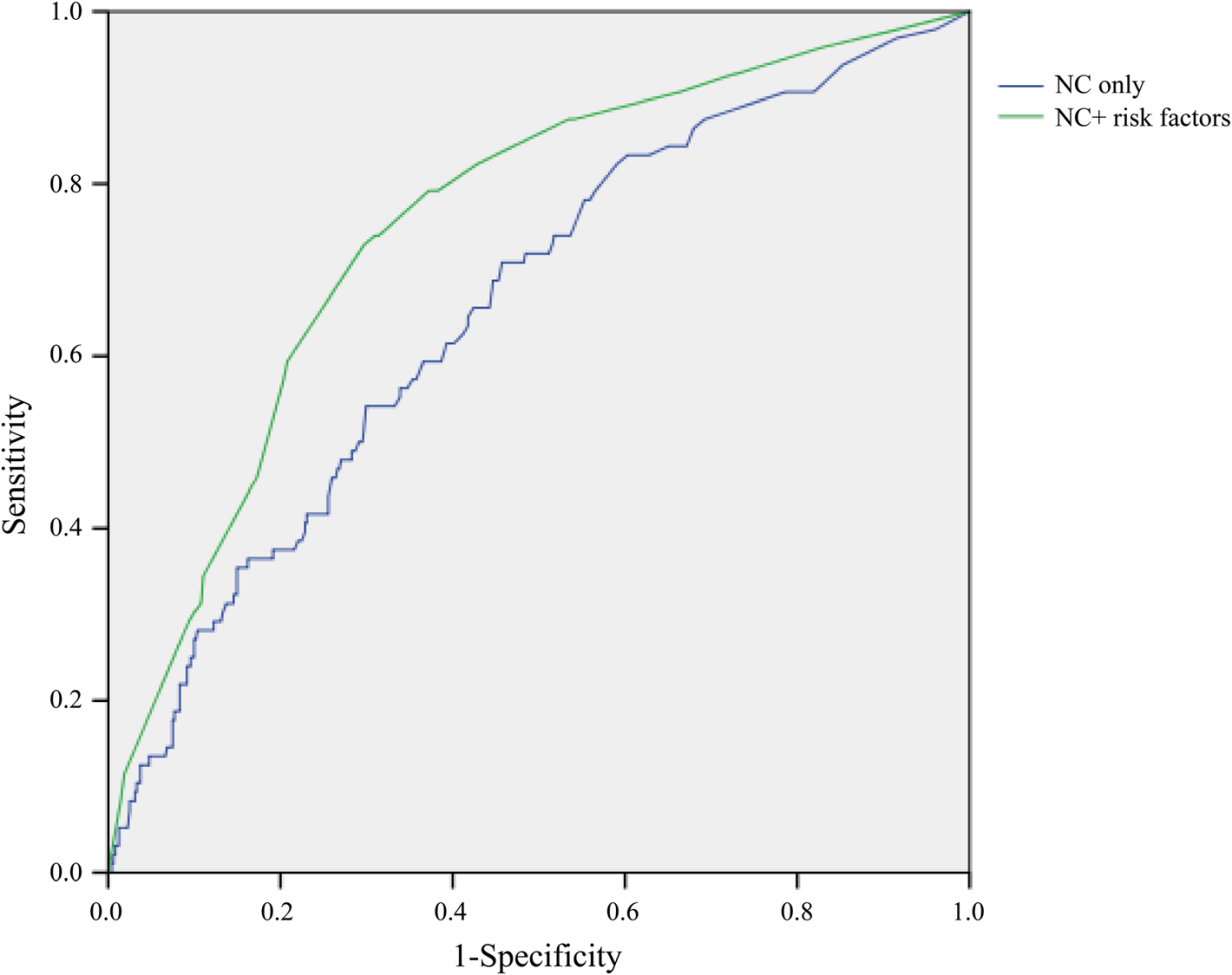
The ROC curves show incorporation of clinic risk factors augments the predictive performance of the NephroCheck. NC only: NephroCheck (+); NC+ risk factors: age > 65 years, CKD, NephroCheck (+) and PCT (+)

Compared with NephroCheck (+) only (AUC = 0.66, 95% CI:0.60–0.72), the combination of NephroCheck (+) and risk factors (age > 65 years, CKD and PCT positive) (AUC = 0.75, 95% CI:0.70–0.81) led to a significant increase in the area under ROC curve for prediction of severe AKI within 7 days (Fig. 3).

## Discussion

AKI is a major complication of major diseases, which is associated with poor outcomes, high mortality and increased resource use [17–19]. The recognition of patients at high risk of developing AKI has attracted more and more attention recently. Early identification of AKI allows for rapid therapeutic intervention to achieve significant clinical benefits. AKI animal models using ischemic, toxic, and septic models have shown that multiple therapeutic agents appear to reduce kidney injury if they are administered before or shortly after injury [20–23].

Cell cycle arrest may be the first process of neurological cell activation upon stress. Protein [TIMP-2] and [IGFBP7] associated with cell cycle arrest are promising markers for the detection of AKI. It has been verified that [TIMP-2] x [IGFBP7] is superior to other biomarkers on detecting AKI in previous cohorts studies [5, 6], which provide early warnings and allow physicians to modify risk, promote intervention and avoid further complications.

Our study demonstrated that NephroCheck is an effective screening tool for recognizing high-risk patients of AKI and it indicates that early NephroCheck (+) is a good predictor of severe AKI. In this study, we found that patients with NephroCheck > 0.3 (ng/ml)^2^/1000 will significantly increase predictive discrimination against subsequent severe AKI. In addition to severe AKI, we also observed that patients with NephroCheck (+) were more likely to have other poor outcomes, such as CRRT initiation and death. Furthermore, as the level of NephroCheck increases, the risk of severe AKI and its poor outcomes also increase. NephroCheck≥2(ng/ml)^2^/1000 patients had highest incidence of severe AKI, CRRT initiation and ICU mortality within 7 days.

Although NephroCheck is a good tool for recognizing the increased risk of AKI and its poor outcomes, indiscriminate biomarker measurement in every patient, regardless of age and comorbidities, will render any test virtually useless [24]. Therefore, integration of clinical risk factors with AKI biomarkers (kidney troponin) can increase sensitivity and discriminate power to kidney attacks [25]. In our study, we have determined predictors of severe AKI, including age > 65 years, CKD, NephroCheck (+) and PCT (+). Some risk factors identified for AKI in our study are consistent with previous literature reports, including age and CKD [13, 16, 17, 26, 27]. The epidemiological association between CKD and AKI makes CKD a risk factor for AKI [13, 17]. Among various hypotheses, it is assumed that patients with CKD have lost renal self-regulation and hemodynamic stability, which explains the small changes in SCr level and the predisposition to subsequent damage [11].

PCT is a predictor of severe AKI in our study. PCT is closely related to the severity of systemic inflammation and bacterial infection [28]. High PCT levels have been considered a good diagnostic indicator of poor prognosis in sepsis patients [29]. Increased PCT levels in patients with pancreatitis and contrast-induced AKI are associated with the development of AKI [30, 31]. Nie et al. [32] reported that PCT can be used as a predictor of AKI for infective patients.

In this study, we found that combination with biomarker NephroCheck and risk factors for risk assessment of AKI has the greatest significance to patients with uncertain disease trajectories. Future research needs to clinically or electronically identify patients at high risk of AKI development or progression to CKD or ESRD [1, 33]. We plan to develop an electronic alert system including the assessment of NephroCheck and risk factors. This risk prediction tool can automatically detect high-risk patients of AKI and help perform early management and individualized treatment of AKI [13, 34–38]. For example, patients identified to suffer high-risk AKI don’t need to wait for the development of AKI, but can begin to optimize volume, adjust drug dose and avoid potential nephrotoxicity based on their AKI risk profile. The aim of the tool is to reduce the severity of AKI and decrease the number of patients requiring dialysis.

Our research also has limitations. First, our study has a small number of severe AKI events. It should be noted that we have excluded 102 patients who had already developed moderate or severe AKI on admission. All of these may reduce the morbidity of AKI. Second, we were unable to determine all risk variables associated with AKI, such as insufficient blood volume, proteinuria, bilirubinuria and exposure to nephrotoxic drugs and contrast media. Furthermore, when a family member is unable to provide a medical history, it is not always possible to determine the chronic risk factors for a comatose ICU patient. Third, the urine criterion was not applied to the diagnosis of AKI in the patient population, as urine output data were not uniformly available. This may have decreased the overall incidence of AKI diagnosis. Finally, by studying NephroCheck as a biomarker in all patients admitted to the ICU, we had a larger sample size. However, this was at the expense of not exclusively focusing on those at risk of AKI. Additionally, biomarkers can only be effectively used in an appropriate clinical context and should be used in high-risk patients of AKI to avoid excessive clinical use.

## Conclusions

In this study, we have determined predictors of severe AKI, including age > 65 years, CKD, NephroCheck (+) and PCT (+). Furthermore, we found that combination with biomarker NephroCheck and risk factors (age > 65 years, CKD and PCT) for risk assessment of AKI has the greatest significance to patients with uncertain disease trajectories.

## Data Availability

The datasets used and analyzed during the current study are available from the corresponding author on reasonable request.

## Abbreviations

AKI: Acute kidney injury
ICU: Intensive care unit
KDIGO: Kidney Disease: Improving Global Outcomes
IGFBP7: Insulin-like growth factor-binding protein 7
TIMP-2: Tissue inhibitor of metalloproteinase-2
SCr: Serum creatinine
FDA: Food and Drug Administration
CRRT: Continuous renal replacement therapy
LOS: Length of stay
SAPS II: Simplified acute physiology score II
MAP: Mean arterial pressure
PCT: Procalcitonin
DM: Diabetes mellitus
CKD: Chronic kidney disease
ESRD: End stage renal disease
CVD: Cardiovascular disease
ROC: Receiver operating characteristic
